# Preliminary Study on the Role of *TMEM39A* Gene in Multiple Sclerosis

**DOI:** 10.1007/s12031-017-0921-1

**Published:** 2017-04-25

**Authors:** Marta Wagner, Maciej Sobczyński, Małgorzata Bilińska, Anna Pokryszko-Dragan, Małgorzata Cyrul, Piotr Kuśnierczyk, Monika Jasek

**Affiliations:** 1 Department of Clinical Immunology, Laboratory of Immunogenetics and Tissue Immunology, Hirszfeld Institute of Immunology and Experimental Therapy, Polish Academy of Sciences, ul. Weigla 12, 53-114 Wrocław, Poland; 2 Department of Genomics, Faculty of Biotechnology, University of Wrocław, ul. Fryderyka Joliot-Curie 14a, 50-383 Wrocław, Poland; 3 Department and Clinic of Neurology, Wroclaw Medical University, ul. Borowska 213, 50-566 Wrocław, Poland

**Keywords:** Multiple sclerosis, *TMEM39A*, mRNA expression, Methylation, Polymorphism

## Abstract

Genome-wide association studies (GWAS) have identified hundreds of new potential genetic risk *loci* associated with numerous complex diseases such as multiple sclerosis (MS). Genes which have been discovered by GWAS are now the focus of numerous ongoing studies. The goal of this study was to confirm and understand the potential role of one of such genes—transmembrane protein 39A gene (*TMEM39A*)—in multiple sclerosis.

We showed the difference in *TMEM39A* messenger RNA (mRNA) expression between MS patients and controls (*T* ^2^_2;74_ = 5.429; *p* = 0.0063). In our study, the lower mRNA expression of *TMEM39A* gene in patients did not correlate with a higher methylation level of the *TMEM39A* promoter. Moreover, a decreased level of *TMEM39A* mRNA was associated neither with rs1132200 nor with rs17281647. Additionally, we did not find an association between these two *TMEM39A* polymorphisms and the risk and progression of multiple sclerosis.

Our investigation is the first which indicates that *TMEM39A* mRNA expression may be associated with the development and/or course of multiple sclerosis.

**Electronic supplementary material:**

The online version of this article (doi:10.1007/s12031-017-0921-1) contains supplementary material, which is available to authorized users.

## Introduction

Multiple sclerosis (MS) is a chronic disease of the central nervous system leading to demyelination and axonal damage in the brain and the spinal cord (Pender and Greer 2007). It is estimated that the disease affects around 2.5 million people worldwide. The most common course of the disease (observed in about 85% patients) is relapsing–remitting (RR) MS, characterized by episodes of acute worsening followed by a recovery period. In the majority of the patients suffering from RR MS, the disease evolves into a secondary progressive (SP) phase characterized by a steady increase in disability without relapses. About 10–15% of patients experience primary progressive (PP) form of MS which is defined by the steady progression from the onset of the disease (Hoffmann and Meinl 2014; Inglese 2006).

Despite the ongoing studies, the etiology and pathogenesis of MS is still not well-defined. However, most scientists concur with the fact that the susceptibility to MS is determined by a combination of environmental and genetic factors. In recent years, thanks to the genome-wide association studies (GWAS), a large number of new genetic risk *loci* for MS such as *IL7R*, *IL2RA*, *CLEC16A*, *CD58*, *EVI5*, *TYK2*, etc. have been identified. Genes discovered by GWAS are now the focus of numerous ongoing studies which goal is to understand their potential role in MS susceptibility (International Multiple Sclerosis Genetics C 2010).

In our study, we concentrated on the transmembrane protein 39A gene (*TMEM39A*), which was found to be associated with MS susceptibility in GWAS performed by International Multiple Sclerosis Genetics Consortium (IMSGC) (International Multiple Sclerosis Genetics C, 2010). Moreover, *TMEM39A* seems to be a susceptibility *locus* for systemic lupus erythematosus (Lessard et al. 2012; Sheng et al. 2015). Recent literature data suggest also that *TMEM39A* may serve as a novel diagnostic marker of gliomas and other cancers (Park et al. 2017). However, to date, very little is known about this gene and its function. Therefore, we decided to perform a preliminary functional study on *TMEM39A* and its role in MS. First, we investigated *TMEM39A* messenger RNA (mRNA) expression level in the group of 39 RR MS patients and 40 controls and then we studied *TMEM39A* methylation status. Moreover, we examined the effect of two *TMEM39A* polymorphisms (rs1132200 and rs17281647) on its mRNA level. Rs1132200 was indicated in literature as being associated with susceptibility to multiple sclerosis (International Multiple Sclerosis Genetics C, 2010) while rs17281647 polymorphism was selected on the basis of in silico analysis as Tag SNP that covers one of the linkage disequilibrium (LD) blocks of *TMEM39A*. Additionally, we conducted a case–control study (on 336 patients and 322 controls) to find a possible association of the investigated polymorphisms with susceptibility and/or progression of MS in a well-defined Polish population. Furthermore, in our study, we also took into account the presence of HLA-DRB1*15:01 allele.

## Materials and Methods

### Study Population

Peripheral blood samples from 39 RR MS patients (26 women and 13 men) with clinically definite MS according to the McDonald criteria (Polman et al. 2011) who did not have immunomodulatory therapy for at least 3 months were collected for mRNA isolation and *TMEM39A* expression analysis. Degree of disability and the rate of its progression were scored using Kurtzke’s Expanded Disability Status Scale (EDSS) and MS Severity Score (MSSS), respectively (Kurtzke 1983; Roxburgh et al. 2005). The controls consisted of 40 volunteers (27 women and 13 men) with no history of inflammatory disease.

Methylation analysis and genotyping of two *TMEM39A* polymorphisms (performed in order to determine their influence on mRNA expression) were carried out on DNA samples from peripheral blood of the same 39 RR MS patients and 40 controls.

In the expanded study, concerning the association of *TMEM39A* polymorphisms with MS susceptibility and course of the disease, 336 patients (226 females and 110 males) were included. Two hundred fifty patients presented relapsing–remitting course of multiple sclerosis, while 86 had secondary progressive form of the disease. We did not include patients with primary progressive course in this study, since we did not collect sufficient number of samples to perform a powerful statistical analysis. Detailed patient characteristics are presented in our earlier publication (Wagner et al. 2014a). Controls were 322 blood donors (138 females and 184 males) with no history of inflammatory disease.

All the patients were under the charge of Department of Neurology, Wroclaw Medical University. The study was approved by the Commission of Bioethics at Wroclaw Medical University and written informed consent was obtained from all individual participants included in the study.

### RNA Isolation and mRNA Expression Analysis

Whole blood samples were collected in PAXgene Blood RNA Tubes. Total RNA was extracted using the PAXgene Blood RNA Kit (Qiagen). The reverse transcription reaction of 500 ng of total RNA was carried out with iScript Reverse Transcription Kit (BioRad) according to the manufacturer’s protocol.

mRNA expression was analyzed by qPCR using 5× HOT FIREPol EvaGreen qPCR Mix Plus (Solis BioDyne). Hypoxanthine phosphoribosyltransferase (*HPRT*) and glyceraldehyde 3-phosphate dehydrogenase (*GAPDH*) were used as reference genes. The primers used in this experiment are listed in Supplementary Table 1. Real-time PCR was performed in a final reaction mixture of 20 μl containing 1.0 μl of cDNA (25 ng/μl). The amplification conditions were as follows: 95 °C for 15 min, (95 °C for 20 s, 61 °C for 30 s, 72 °C for 40 s) × 40 cycles. All samples were run in triplicate. In order to verify the reproducibility of results, the calibrator sample was run on each plate.

### Methylation Analysis—Methylation-Sensitive High-Resolution Melting (MS HRM)

Genomic DNA was isolated from whole blood using Invisorb Blood Midi Kit (Stratec Molecular) according to the manufacturer’s protocol.

EZ DNA Methylation-Gold Kit (Zymo Research) was used for bisulfite conversion of the DNA. The starting amount of DNA was 1 μg. All the modification reactions were performed according to the manufacturer’s protocol.

PCR amplification followed by high-resolution melting analysis was carried out on ViiA7 Real-Time PCR System (Thermo Fisher Scientific). PCR was performed in a final reaction mixture of 20 μl containing the following: 1× MeltDoctor HRM Master Mix, 250 nM of each primer (please see Supplementary Table 1), 1.5 mM MgCl_2_, and 10 ng of bisulfite-converted template. Primers were designed according to the protocol published by Wojdacz et al. (Wojdacz et al. 2008). The amplification conditions were as follows: 95 °C for 10 min, (95 °C for 15 s, 60 °C for 1 min) × 40. HRM analysis was performed using the following conditions: 95 °C for 10 min, 60 °C for 1 min, 95 °C for 15 s, and 60 °C for 1 s. All reactions were run in triplicate. Non-bisulfite-treated DNA was used as a negative control to ensure no amplification of genomic DNA. Methylation status of the examined samples was determined by comparing their melting profiles with the profiles derived from dilution series (0, 1, 10, 25, 50, 75, and 100%) of the methylated and unmethylated control template (Human Methylated and Non-methylated DNA Set, Zymo Research). The normalized melting curves for each standard are shown in Supplementary Figure 1.

### Genotyping

The examined SNPs of *TMEM39A* were genotyped by applying polymerase chain reaction followed by restriction fragment length polymorphism (PCR-RFLP). Supplementary Table 1 shows primer sequences, annealing temperatures, and restriction enzymes used in this study.

HLA-DRB1*15:01 status was determined in our earlier studies (Wagner et al. 2013; Wisniewski et al. 2013) as described by Wisniewski et al. (Wisniewski et al. 2013), and these data were taken into account in the analyses performed here.

Accuracy of genotyping methods for the investigated SNPs was verified through direct sequencing of few samples representing homozygotes of two types and heterozygotes for each investigated SNP. These samples were used as the reference samples in following genotyping experiments.

### Statistical Methods

*TMEM39A* mRNA expression (measured as 2^−ΔCt^) normalized to *HPRT* and to *GAPDH* was described by median, 1st (Q1), and 3rd (Q3) quartiles as well as by the minimal and maximal observation. In case of median,*S*_*n*_statistic was computed as the measure of variability: *S*_*n*_ = med{med|*x*_*i*_ − *x*_*j*_|; *j* = 1 … *n*} (Rousseeuw and Croux 1993).*S*_*n*_ is a typical difference between two randomly selected observations. Chi-square, χ^2^, test was used to test the hypothesis that two groups have the same distribution of genotype counts. When the sample sizes were small, distributions of the test statistics were estimated numerically. Odds ratio (OR) and confidence interval for them at 1-α = 0.95 (CI 95%) were computed as the measures of effect size. We assumed additive model of association between genotype and the risk of MS. *TMEM39A* mRNA expression, normalized to *HPRT* and *GAPDH*, was modeling as two-dimensional variable*x*_*i*_ = (HPRT_*i*_, GAPDH_*i*_) ∈ *ℜ* ^2^and significances was tested with *λ* Pillai and *T* ^2^ Hotelling statistics. Similarly, MS progression and its association with genetic factors was modeling as*x*_*i*_ = (EDSS_*i*_, MSSS_*i*_) ∈ *ℜ* ^2^. Differences in the age of onset dependent on genetic factors were tested with analysis of variance F-statistic. Duration of the relapsing–remitting course was analyzed with the proportional hazards model. Haplotype frequencies were estimated with the *maximum likelihood* function (Excoffier and Slatkin 1995). Likelihood ratio statistic LRS_*df* = 3_ = 2(*LL*_Cases_ + *LL*_Controls_ − *LL*_Combined_) was used to test the differences in haplotype frequencies between cases and controls. Departure from Hardy–Weinberg equilibrium (HWE) was measured as $f=\frac{p_{C C}-{p}_C^2}{p_C\left(1-{p}_C\right)}$, where *p*_*C*_and*p*_*CC*_ are allele *C* and genotype *CC* frequencies. *f* < 0 in case of deficiency of homozygotes, *f* > 0 corresponds to deficiency of heterozygotes, and *f* = 0 when *locus* is in HWE. Linkage disequilibrium (LD) was measured with *r* ^2^ statistic. Results were considered as statistically significant at *p* < 0.05.

## Results

### TMEM39A mRNA Expression in Patients and Controls

**Fig. 1:**
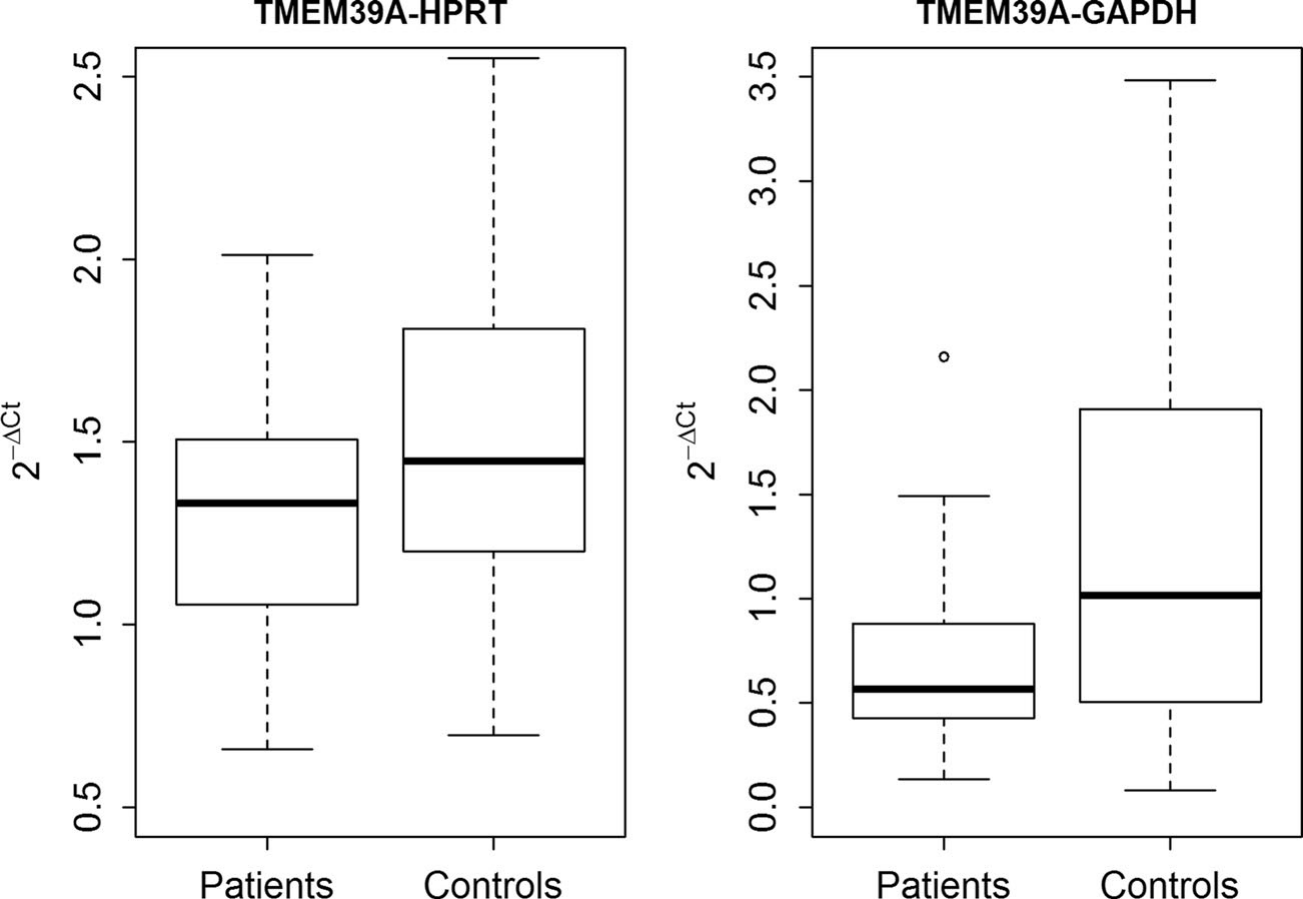
Difference in *TMEM39A* mRNA expression between MS patients and controls, *T* ^2^_2;74_ = 5.429; *p* = 0.0063. Thirty-nine MS patients with relapsing-remitting course of the disease and 40 controls were included in *TMEM39A* expression analysis. *TMEM39A* mRNA expression was normalized to *HPRT* and *GAPDH* and measured as 2^−ΔCt^. Box-and-whiskers plot presents the median expression of *TMEM39A* gene in controls and patients, 1st and 3rd quartiles, minimal and maximal non outlier observation

In the initial phase of our study, the level of *TMEM39A* mRNA expression in MS patients and controls was determined. We were able to observe a lower expression of *TMEM39A* mRNA in patients than in healthy individuals (*T* ^2^_2;74_ = 5.429; *p* = 0.0063) (Supplementary Table 2). Figure 1 shows *TMEM39A* mRNA expression normalized to two reference genes—*HPRT* and *GAPDH*.

### Methylation Level of TMEM39A Gene

Since methylation plays an important role in the regulation of gene expression and since in silico analysis revealed CpG islands in the promoter region of *TMEM39A* gene (Li and Dahiya 2002), in the next step, we decided to check whether a lower expression of mRNA is correlated with a higher level of methylation of this gene. We were not able to find such relationship. In fact, we observed that *TMEM39A* gene was unmethylated both in controls and patients (Supplementary Figure 1B).

### TMEM39A mRNA Expression in Dependence of Polymorphisms rs17281647 and rs1132200

Next, we wanted to find out whether the expression of *TMEM39A* mRNA is dependent on genotype at rs17281647 and rs1132200 polymorphic sites. We also took into consideration the presence of HLA-DRB1*15:01 allele (the most consistently replicated genetic risk factor for MS) and gender. The results showed that the analyzed variables were not associated with *TMEM39A* mRNA expression (in combined group of patients and controls). Moreover, the lower expression of *TMEM39A* mRNA in MS patients was independent of these variables (*F*_df=16;138_ = 0.564; *p* = 0.906).

### Polymorphisms vs MS Susceptibility and Clinical MS Data

Additionally, we expanded the investigation of two *TMEM39A* polymorphisms (rs1132200 and rs17281647) in the group of 336 patients and 322 controls in order to find the possible association between these two SNPs and susceptibility and/or progression of MS.

For polymorphism rs17281647, we found no evidence for deviation from Hardy–Weinberg equilibrium both in controls and cases (χ^2^ = 0.149; *p* = 0.713 and χ^2^ = 2.642; *p* = 0.115, respectively). Similarly, rs1132200 was in HWE in controls (χ^2^ = 1.394; *p* = 0.271), however in the patient group, deviation from HWE was observed (χ^2^ = 6.14; *p* = 0.021). In this group, we noticed the overrepresentation of GA heterozygotes (*f* = −0.135 CI 95% (−0.19; −0.06)).

Our study revealed that neither of the SNPs analyzed by us was associated with MS risk in our population (Supplementary Table 3). Since in our case–control study HLA-DRB1*15:01 allele was strongly associated with MS (for HLA-DRB1*15:01−/+ OR = 2.66; 95% CI = 1.86–3.75; *p* < 0.0001 and for HLA-DRB1*15:01+/+ OR = 4.34; 95% CI = 1.61–12.73; *p* = 0.006) (Wagner et al. 2014b) and stratification for HLA-DRB1*15:01 is expected to help to reveal the associations with MS susceptibility (Bronson et al. 2010; Kikuchi et al. 2002), the presence of this allele was also taken into consideration. However, the lack of association of genotypes in rs17281647 as well as in rs1132200 with susceptibility to multiple sclerosis was found in both HLA-DRB1*15:01– and HLA-DRB1*15:01+ individuals (data not shown).

In the next phase, we analyzed the haplotype frequency in both the controls and the patients. We did not observe any differences in their distribution between the compared groups (LL_df=3_ = 6.308; *p* = 0.098).

The analysis of linkage disequilibrium showed that the investigated SNPs were not in LD with each other in controls as well as in patients (*r* ^2^ = 0.018 and *r* ^2^ = 0.038, respectively).

Since it was hypothesized that genes associated with susceptibility to MS (including *TMEM39A*) may also determine various features of its phenotype (Mowry et al. 2013), we examined the possible association between polymorphisms rs17281647 and rs1132200 and available clinical data. We observed that neither of them was associated with duration of relapsing–remitting course of the disease (χ^2^_df=2_ = 1.764; *p* = 0.414). This result was adjusted to MSSS, EDSS, and age of onset, which were associated with duration of RR course of MS, as we showed in our previous study for *ALCAM* gene (Wagner et al. 2013). Furthermore, neither rs17281647 nor rs1132200 was associated with disease progression measured on the disability scales—EDSS and MSSS (*λ*_Pillai_ = 0.013; *p* = 0.377). We also found no evidence for association between *TMEM39A* polymorphisms and age of onset (*F*_df=3;222_ = 2.416; *p* = 0.091).

## Discussion

The results of the study performed by IMSGC, presenting *TMEM39A* (3q13.33) as potential MS susceptibility *locus* (International Multiple Sclerosis Genetics C 2010) prompted us to investigate this gene in well-defined Polish population. According to our knowledge, this is the first study to report evidence of different *TMEM39A* mRNA levels in MS patients and controls. Since, to date, very little is known about TMEM39A gene, it is hard to predict what role it may play with regard to multiple sclerosis. As stated in GeneCards database (www.genecards.org), TMEM39A may be involved in positive regulation of antiviral response by host. Since a large body of evidence indicates that infection with the Epstein–Barr virus has a major role in the pathogenesis of MS (Pender and Burrows, 2014), one might expect that lower expression of TMEM39A mRNA (assuming positive correlation between the levels of mRNA and protein) may be associated with higher susceptibility to infection with Epstein–Barr virus and as a consequence with higher susceptibility to multiple sclerosis. However, further studies are necessary to verify this hypothesis, to find out in what way lower expression of this gene may influence the susceptibility to MS and what mechanism is responsible for decreased *TMEM39A* mRNA expression.

One of the mechanisms which may cause silencing of the genes, possibly by blocking the binding of transcriptional factors to promoters, is DNA methylation. Recent evidence suggests the role of epigenetics (DNA methylation, histone modifications, and microRNA (miRNA)) in multiple sclerosis (Kucukali et al. 2015). Methylation status of several genes has been found to be altered in peripheral blood mononuclear cells (PBMC) and/or brain samples from MS patients as compared to those from healthy donors (Kucukali et al. 2015). The *SHP-1* gene may serve as an example. The considerably reduced SHP-1 activity in PBMC of MS patients was noticed (Christophi et al. 2008) and it was associated with increased *SHP-1* promoter methylation (Kumagai et al. 2012). This modification presumably results in increased proinflammatory gene expression and, as a consequence, in leukocyte-mediated inflammation (Kucukali et al. 2015). Taking the above into account, we decided to study the methylation level of *TMEM39A* promoter. Unfortunately, we were not able to observe higher methylation level correlated with lower *TMEM39A* expression in MS patients. In fact, we noticed that *TMEM39A* promoter was unmethylated both in controls and patients. It has to be mentioned that our study on the methylation of *TMEM39A* gene had preliminary status and further investigation of other putative functional methylation *loci* is necessary.

Taking into consideration the fact that altered mRNA expression may be caused by genetic variations, next, we decided to examine the potential influence of two *TMEM39A* polymorphisms (rs1132200 and rs17281647) on gene expression. Rs1132200 was indicated in the literature as being associated with susceptibility to multiple sclerosis (International Multiple Sclerosis Genetics C 2010; Varade et al. 2012) as well as systemic lupus erythematosus (Lessard et al. 2012; Sheng et al. 2015). This SNP causes non-synonymous amino acid change (Ala-Thr) at position 487 in the protein. However, it is not known, if it is a functional variant itself or if it is a marker for a different functional variant. The second polymorphism analyzed in our study, rs17281647, was selected on the basis of in silico analysis. In accordance with SNPinfo Web Server (Xu and Taylor 2009), rs17281647 is Tag SNP that covers one of the LD blocks of *TMEM39A*. Although rs17281647 is located in the intronic region, it is in strong LD with rs2282175 polymorphism located in the potential binding site for transcription factor, which may potentially alter the expression of *TMEM39A*. In our study, neither rs17281647 nor rs1132200 was associated with *TMEM39A* mRNA expression. On the one hand, lack of such association may be caused by the low number of samples analyzed in this study (since only patients who did not have immunomodulatory therapy for at least 3 months were taken into consideration). On the other hand, different (than those analyzed here) genetic variants or different mechanisms may underlie lower *TMEM39A* mRNA expression in MS patients.

Further studies are necessary to confirm lower expression of *TMEM39A* mRNA in MS patients and to find the mechanism which may be responsible for the decreased level of *TMEM39A* mRNA in MS patients. Since the expression of *TMEM39A* mRNA was measured in PBMC, it would be also essential to establish in further studies whether the lower level of *TMEM39A* mRNA is not the result of different proportion of subpopulation of cells expressing TMEM39A in samples from MS patients and controls and to check the correlation between mRNA and protein level.

Next, we decided to expand our study of *TMEM39A* polymorphisms into the group of 336 patients and 322 controls. We did not find an association of rs1132200 with MS susceptibility. In contrast to our findings, GWAS conducted by IMSGC (International Multiple Sclerosis Genetics C 2010) as well as replication studies performed on a Spanish population (Varade et al. 2012) and Indian population (D'Cunha et al. 2016) identified polymorphism rs1132200G>A of *TMEM39A* as the potential risk factor for MS with the common allele (G allele) increasing predisposition. According to Salanti et al. (Salanti et al. 2005), in the presence of an association, Hardy–Weinberg equilibrium in cases does not have to be maintained. Moreover, it was proposed that screening with HWE of data sets of affected individuals may be a relatively efficient method for detecting gene–disease associations (Salanti et al. 2005). Therefore, we presume that deviation from HWE observed by us in the group of patients (χ^2^ = 6.14; *p* = 0.021) may indicate the association of this polymorphism with MS also in our population and that the lack of significant difference in genotypes distribution between MS cases and controls may be caused by weak effect of this polymorphism on MS risk. Our study achieved 26% power to detect OR ≤0.79 (or equivalently OR ≥1.26) and 80% power to detect OR ≤0.63 (equivalently OR ≥1.59). It will be important to establish in follow-up studies on a larger population whether A allele in rs1132200 is also the protective allele in the Polish population.

As we mentioned earlier, the second SNP analyzed here (rs17281647) is the intronic variant, however being in full LD (*r* ^2^ = 1.0 according to SNPinfo Web Server) with polymorphism rs2282175 located in potential binding site for transcription factor. Moreover, the results of in silico analysis showed that it is also in full LD with rs1132202 SNP, which is located in the putative binding site for miRNA. We did not find the difference in genotype distribution in the analyzed polymorphic site between MS patients and healthy individuals. With our numbers of individuals in control and patient groups, we were able to detect OR ≤0.82 (equivalently OR ≥1.22) with probability *P* = 0.23 (power 23%) and OR ≤0.63 (equivalently OR ≥1.59) with *P* ≥ 0.8 (power 80%). Since this is the first study which investigated the possible association of rs17281647 polymorphism with susceptibility to MS and course of the disease and because it achieved limited power to detect weak associations, further studies on a larger group are necessary.

Furthermore, we did not observe an association between rs1132200 and rs17281647 polymorphisms and progression of the disease.

In conclusion, although we were not able to find the association of *TMEM39A* polymorphisms with predisposition to multiple sclerosis and course of the disease, we showed that its mRNA expression is decreased in PBMC of MS patients.

## Electronic supplementary material

Supplementary Figure 1 (DOCX 418 kb)

Supplementary Table 1 (DOCX 17 kb)

Supplementary Table 2 (DOCX 17 kb)

Supplementary Table 3 (DOCX 19 kb)
