# Supplementary material for: Preliminary Study on the Role of TMEM39A Gene in Multiple Sclerosis
Source: J Mol Neurosci. 2017 Apr 25;62(2):181–7. doi: 10.1007/s12031-017-0921-1 (PMC5486520; doi:10.1007/s12031-017-0921-1)
Supplement: Supplementary file 1 — (DOCX 418 kb) [file 12031_2017_921_MOESM1_ESM.docx]

Supplementary Figure 1A. The normalized melting-curves for each standard.

Supplementary Figure 1B. The normalized melting-curves for each standard together with melting curves for a few samples.

100% methylated DNA was mixed in different ratios with non-methylated DNA to obtain 0%, 1%, 10% , 25%, 50%, 75% and 100% methylated standards. The curves for standards and for examined samples were generated with the same pair of primers which amplify a 141 bp PCR fragment of *TMEM39A* containing 12 CpG dinucleotides.
