# Supplementary material for: Preliminary Study on the Role of TMEM39A Gene in Multiple Sclerosis
Source: J Mol Neurosci. 2017 Apr 25;62(2):181–7. doi: 10.1007/s12031-017-0921-1 (PMC5486520; doi:10.1007/s12031-017-0921-1)
Supplement: Supplementary file 2 — (DOCX 17 kb) [file 12031_2017_921_MOESM2_ESM.docx]

Supplementary Table 1. Primer sequences, annealing temperatures and restriction enzymes used in the present study.

| **Genotyping** | | | | | | |
| --- | --- | --- | --- | --- | --- | --- |
| **Polymorphism** | **Forward primer** | **Reverse primer** | | | **T_a_** | **Restriction**  **enzyme** |
| **rs17281647** | TGCTGGCTTGGCTAAATAAG | ACCATGCTTTCCACAAACAA | | | 60^°^C | TaiI |
| **rs1132200** | CCATGGCTCTCATCCTCTTC | TTCCCGACTTTCAAAACATAA | | | 62^°^C | MwoI |
| **mRNA expression analysis** | | | | | | |
| **Gene** | **Forward primer** | | | **Reverse primer** | | |
| ***TMEM39A*** | TCAGCTTTACCTTCTTTGCAGAC | | | GACTGGAAGGCCAATAGCAC | | |
| ***GAPDH^a^*** | GAGTCCACTGGCGTCTTCAC | | | TTCACACCCATGACGAACAT | | |
| ***HPRT^a^*** | TGACCTTGATTTATTTTGCATACC | | | CGAGCAAGACGTTCAGTCCT | | |
| **Methylation analysis** | | | | | | |
| **Gene** | **Forward primer** | | **Reverse primer** | | | |
| ***TMEM39A*** | CGTTGAGGGTAGGAGGAATAAT | | CGACTCCCGAACCATCTTTATA | | | |

*^a^* Primer sequences were kindly provided by Karolina Marek-Bukowiec, MSc from Laboratory of Tumor Immunology,

Institute of Immunology and Experimental Therapy , Wroclaw
