# Supplementary material for: Preliminary Study on the Role of TMEM39A Gene in Multiple Sclerosis
Source: J Mol Neurosci. 2017 Apr 25;62(2):181–7. doi: 10.1007/s12031-017-0921-1 (PMC5486520; doi:10.1007/s12031-017-0921-1)
Supplement: Supplementary file 3 — (DOCX 17 kb) [file 12031_2017_921_MOESM3_ESM.docx]

Supplementary Table 2. *TMEM39A* mRNA expression (measured as 2^-ΔCt^) normalized to *HPRT* and *GAPDH*

|  | Min | Q1 | Median | S_n_ | Q3 | Max |
| --- | --- | --- | --- | --- | --- | --- |
| ***HPRT - normalized*** | | | | | | |
| **Patients** | 0.6579 | 1.0550 | 1.333 | 0.2771 | 1.5070 | 2.013 |
| **Controls** | 0.6979 | 1.2100 | 1.448 | 0.3559 | 1.8050 | 2.552 |
| ***GAPDH - normalized*** | | | | | | |
| **Patients** | 0.1355 | 0.4280 | 0.5655 | 0.2798 | 0.8796 | 2.158 |
| **Controls** | 0.0833 | 0.5114 | 1.0320 | 0.8356 | 2.0360 | 4.006 |

Min, Max – minimal and maximal value of 2^-ΔCt^

Q1 – first quartile

S_n_ – measure of variability; higher value – higher variability

Q3 – third quartile
