# Supplementary material for: Preliminary Study on the Role of TMEM39A Gene in Multiple Sclerosis
Source: J Mol Neurosci. 2017 Apr 25;62(2):181–7. doi: 10.1007/s12031-017-0921-1 (PMC5486520; doi:10.1007/s12031-017-0921-1)
Supplement: Supplementary file 4 — (DOCX 19 kb) [file 12031_2017_921_MOESM4_ESM.docx]

Supplementary Table 3. Genotype distribution of two *TMEM39A* polymorphisms in multiple sclerosis patients and controls.

| ***TMEM39A rs17281647 G>A*** | | | | | | | |
| --- | --- | --- | --- | --- | --- | --- | --- |
|  | **Cases** | | **Controls** | | **OR** | **CI95%** |  |
|  | **N** | **%** | **N** | **%** |  |  |  |
| **GG** | 229 | 68.1 | 233 | 72.4 | 1^a^ |  | χ^2^_df=1_= 2.306;  p = 0.129 |
| **GA** | 98 | 29.2 | 86 | 26.7 | 1.16 | 0.82; 1.63 |  |
| **AA** | 9 | 2.7 | 3 | 0.9 | 3.05 | 0.81; 11.41 |  |
| **∑** | 336 | 100.0 | 322 | 100.0 |  |  |  |
| **GG** | 229 | 68.1 | 233 | 72.4 | 1^a^ |  | χ^2^_df=1_= 1.388;  p = 0.239 |
| **GA+AA** | 107 | 31.9 | 89 | 27.6 | 1.22 | 0.87; 1.71 |  |
| ***TMEM39A rs1132200 G>A*** | | | | | | | |
|  | **Cases** | | **Controls** | | **OR** | **CI95%** |  |
|  | **N** | **%** | **N** | **%** |  |  |  |
| **GG** | 235 | 69.9 | 240 | 74.5 | 1 ^a^ |  | χ^2^_df=1_=0.390;  p=0.532 |
| **GA** | 99 | 29.5 | 73 | 22.7 | 1.38 | 0.97; 1.96 |  |
| **AA** | 2 | 0.6 | 9 | 2.8 | 0.23 | 0.05; 1.08 |  |
| **∑** | 336 | 100.0 | 322 | 100.0 |  |  |  |
| **GG** | 235 | 69.9 | 240 | 74.5 | 1^a^ |  | χ^2^_df=1_=1.726;  p=0.189 |
| **GA+AA** | 101 | 30.1 | 82 | 25.5 | 1.26 | 0.89; 1.77 |  |

^a^ reference group
